# Influence of long -term thermal cycling and masticatory loading simulation on bond strength of roots filled with epoxy resin and calcium silicate based sealers

**DOI:** 10.1186/s12903-023-03377-1

**Published:** 2023-09-18

**Authors:** Ahlam Smran, Mariam Abdullah, Norasmatul Akma Ahmad, Ali Alrahlah, Nassr AL-Maflehi, Abdulaziz Samran

**Affiliations:** 1 Department of Restorative Dentistry, Faculty of Dentistry, University of Malaya, Kuala Lumpur, Malaysia; 2 Department of Restorative and Prosthetic Dental Sciences, College of Dentistry, Dar Al-Uloom University, Riyadh, Saudi Arabia; 3 Restorative Dental Sciences Department, Engineer Abdullah Bugshan Research Chair for Dental and Oral Rehabilitation, College of Dentistry, King Saud University, 11545 Riyadh, Saudi Arabia; 4 Periodontics and Community Dentistry, College of Dentistry, King Saud University, Riyadh, Saudi Arabia; 5 Department of Prosthodontics, School of Dentistry, Ibb University, Ibb, Yemen

**Keywords:** AH plus, Calcium silicate sealer, Push out bond strength, Thermal, Mechanical cycling

## Abstract

**Background:**

The aim of this study was to evaluate the effect of thermal and mechanical cyclic aging using a mastication simulator on push-out bond strength of mandibular premolars obturated with AH Plus and BioRoot RCS root canal sealers.

**Methods:**

With REVO-S files up to SU/0.06 taper, 48 single-rooted premolar teeth were instrumented. The teeth were randomly divided into two main groups (*n* = 24) based on the two root canal sealers used (AH Plus and BioRoot RCS). All teeth were obturated with h matched-taper single-cone. Each main group was then subdivided into three subgroups (A, B, and C) (*n* = 8). Group A served as the negative control group (no-thermocycling aging). While groups B and C were subjected to thermal changes in a thermocycler machine (15,000 and 30,000 thermal cycles, respectively), followed by two different dynamic loading periods, 3 × 10^5^ and 6 × 10^5^ in a masticatory simulator with a nominal load of 5 kg at 1.2 Hz which represent roughly 1½ and 3 years of clinical function respectively. 2 mm slice at 3 levels, apical, middle, and coronal, to obtain 3 sections were prepared and subjected to push-out test using a universal testing machine. Statistical analysis was performed using analysis of variance (ANOVA) followed by a Tukey post hoc comparisons test and an independent T-test. A significance level of 5% was used.

**Results:**

After thermal–mechanical cyclic aging, the two root canal sealers showed a significantly decreased in push-out bond strength (*p* < 0.05), however, AH Plus had significantly higher bond strength values than BioRoot RCS after cycling aging.

**Conclusions:**

It could be concluded that thermal–mechanical cyclic aging had a significant impact on the outcome of the dislodgment resistance of AH Plus and BioRoot RCS.

## Background

The main goals of canal space obturation are to seal off any microorganisms that were not completely eliminated during the cleaning and shaping processes and to prevent leakage into the root canal system from the oral cavity and periapical tissues. Additionally, sealers should adhere to dentin, lowering the risk of failure of the endodontic treatment [1, 2]. Endodontically treated teeth (ETT) may have an extended clinical lifetime and improved resistance to fracture as a result of the use of a sealer with increased adhesion to dentin, increasing the strength of the repaired tooth [3].

Predictable clinical results have been associated with the use of gutta-percha (GP) in combination with root canal sealers such as zinc oxide and eugenol or epoxy resin [4]. With low solubility, excellent flow and apical sealing, biocompatibility, and adherence to root dentin, the epoxy resin-based sealer AH Plus (Dentsply-De Trey, Konstanz, Germany) has been considered the standard among endodontic sealers [5]. Nevertheless, there are limitations associated with it, such as cytotoxicity, an inflammatory response, and the potential for mutagenic effects. Moreover, as a result of its hydrophobic nature, the hydrophilic channel cannot achieve full fluid saturation. Dental moisture that remains trapped, specifically, can result in difficulties when it comes to AH Plus adhering to the walls of the canal. [6]. Thus, researchers are constantly looking for improved sealers or root filling materials with higher dislocation resistance and better sealing characteristics. A calcium silicate-based root canal sealer (BioRoot RCS, Septodont, St. Maur-des-Fossés, France) is primarily tricalcium silicate and zirconium oxide powder mixed with a liquid containing calcium chloride. BioRoot RCS releases calcium hydroxide after setting [7] and leaches high levels of calcium [8]. Additionally, it is highly bioactive, inducing the production of angiogenic and osteogenic growth factors and forming a calcium phosphate phase when in contact with physiological solutions [9].

In terms of dental practice, there are primarily two benefits to having the root sealer adhere to the dentinal walls [10]. In a static situation, ideal adhesion resulted in fewer regions with gaps that would permit fluid infiltration at interfaces between sealer and dentin or sealer and core-filling substance. It prevents sealer dislodgment during operational functional processes in a dynamic environment, which raises success rates [11]. Consequently, testing the samples under cyclic loading will be more clinically relevant and will improve predictions of how dental filling materials would perform when used in vivo [12].

Push-out bond strength (POBS), also known as dislodgement resistance, has been recognized as an important prognostic criterion for assessing the connection between a root canal sealer and the canal wall and the core material [13]. A few studies have evaluated the POBS of roots filled with BioRoot RCS [14–18]. However, these studies assessed the bonding effectiveness of adhesives after static bond-strength tests. Under clinical circumstances, It is debatable whether the results of a static analysis have clinical significance because they do not accurately reflect actual occlusal loads and because the stress reactions to dynamic and static loads differ [19]. A previous study [20], used thermocycling process to simulate the physiological aging of endodontic sealers, reported increase of the bond strength of BioRoot RCS with increased thermocycles. It was recently reported that when thermal and mechanical load cycling were performed concurrently, a significant decrease in bond strength of adhesives to dentin was observed when compared to specimens that were thermal cycled or subjected to mechanical loading alone. Perhaps the simultaneous combination of mechanical and thermal cycling (thermal–mechanical cycling) could induce faster mechanical degradation and fatigue of root canal sealers [21]. The authors are unaware of a study evaluating the POBS of teeth obturated with AH Plus or BioRoot RCS that mimicked long-term clinical conditions using two methods of aging.

Therefore, this study aimed to determine whether thermal–mechanical cyclic aging might affect the POBS of roots filled with AH Plus and BioRoot RCS at two different periods (15,000/3 × 10^5^ and 30,000/6 × 10^5^, simulating approximately 1½ and 3 years of clinical function. The null hypothesis was that thermal–mechanical cyclic aging would not affect the POBS of roots filled with AH Plus and BioRoot RCS in relation to time (1½ and 3 years).

## Methods

### Sample size calculation

In this study, the sample size was calculated following the one described by Ahlam et al. [22]. An a-priori power analysis was performed with the G*Power software to determine sample size (G*Power V 3.1.9.7 Franz Faul, Universität Kiel, Kiel, Germany). The purpose of this experiment investigation was to detect statistical significance among groups with an effect size of 0.65 and 82% power at α = 0.05. This computation indicated that 24 removed teeth were required (8 for each group).

### Specimens preparation

**Fig. 1:**
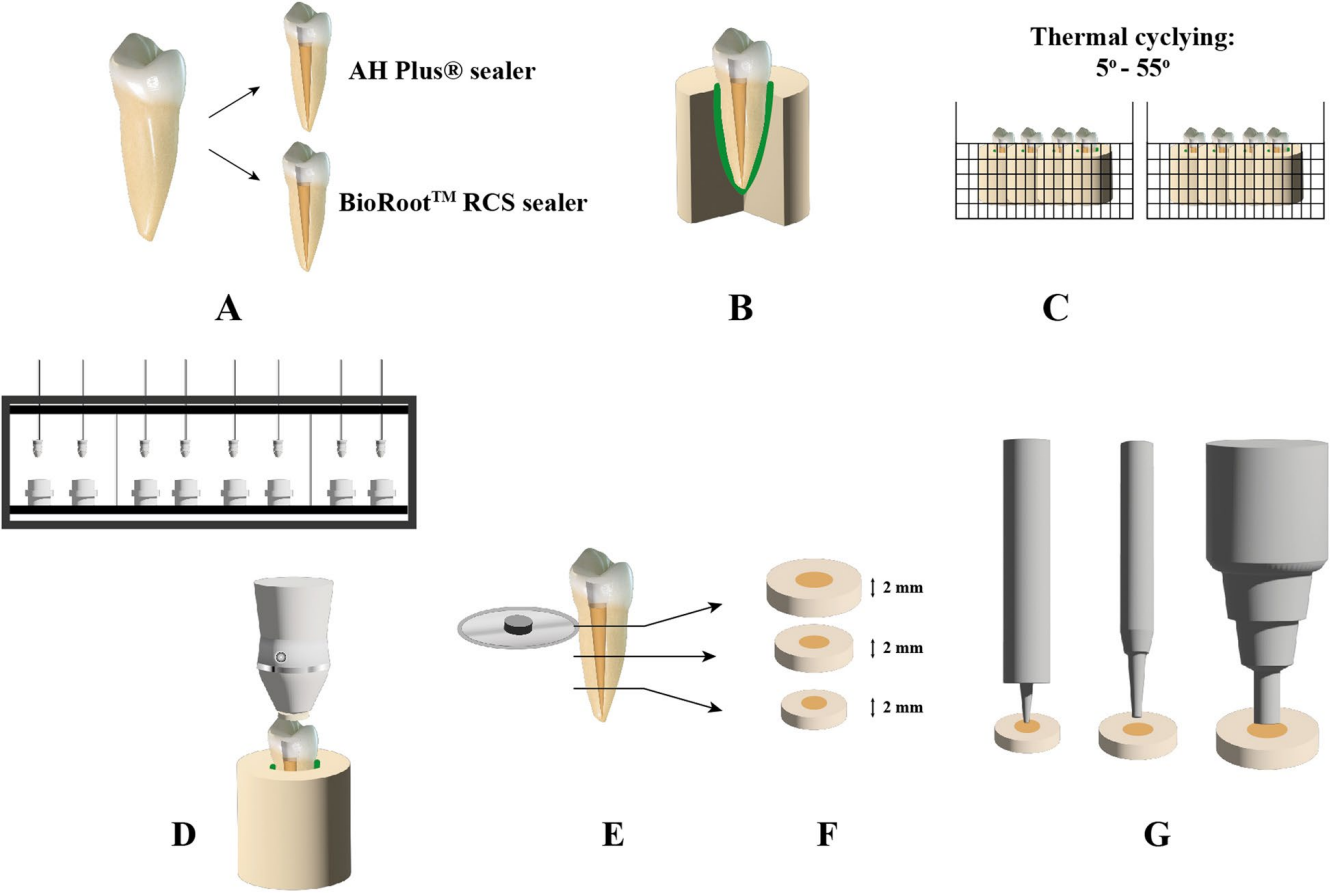
Schematic presentation of prepared specimens and push-out testing. **A** Root canal treatment and canal obturation with tested sealers. **B** Embedding the root in acrylic resin block. **C** Thermal variations in a thermal cycling machine. **D** Mechanical cycling in a mastication simulator machine. **E**, **F** Coronal, middle, and apical root-dentin sections. **G** Load application in universal testing machine using 3 different plunger diameters

Part of this study’s methodology was justified to follow a previous study [22]. The study proposal was reviewed and approved by the Medical Ethics Committee of the Faculty of Dentistry, Malaya University, Malaysia (DF-RD-1912/0032-P). In addition, informed consent was obtained from all participants from whom samples were taken. Schematic presentation of the specimen preparation and push-out testing is presented in Fig. 1. Forty-eight single-rooted mandibular premolars extracted for orthodontic purposes from patients aged 18 to 30 were collected. Teeth with similar dimensions (buccolingually and mesiodistally), and root length of 22 mm (1 mm) from the tip of the buccal cusp were examined to control biological variations using a digital caliper (Measuring Tool Enterprise, Shanghai, China). Teeth with caries, an open apex, or a previous root canal treatment were ruled out. Periapical radiographs in both buccolingual and mesiodistal were taken to establish the presence of a straight single root canal with no calcification or resorption. The selected teeth were stored in a 0.12% thymol solution for three months before being used [22].

The pulp chamber was accessed using a No. 4 round bur (Dentsply Maillefer, Ballaigues, Switzerland). K-file size 10 (MANI Inc, Utsunomiya, Tochigi, Japan) was used to set the working length at 1 mm below the apex. All teeth were instrumented with REVO-S rotary files (MICRO-MEGA, Besancon Cedex, France) up to SU/0.06 taper. After each file, 3 mL of 2.5% NaOCl was used to irrigate the root canals. Following instrumentation, the root canals were washed for 60 s with 1 mL of 17% EDTA and 2 mL of 5.25% NaOCl (Pulpdent Corp., Watertown, MA). The last rinse consisted of 5 mL of normal saline. The canals were dried with paper tips just before obturation (Brasseler USA). The teeth were divided into two groups (*n* = 24) randomly: Group 1: AH Plus + GP (Dentsply Maillefer NA, Tulsa, OK); Group 2: BioRoot RCS + GP (Dentsply Maillefer NA, Tulsa, OK). The root canals were obturated using matched-taper single-cone gutta percha coated with the tested sealers and applied in accordance with the manufacturer's instructions. To verify that the root filling was adequate in terms of length, density, and taper, radiographs of the mesiodistal and buccolingual regions were taken. Composite resin (MultCore Flow; Ivoclar AG, Schaan, Liechtenstein) was used to fill the access opening. The teeth were kept at 37 °C with 100% humidity for 7 days to make sure the root canal sealers had fully set.

### Thermal–mechanical cyclic aging

To replicate the periodontal ligament, the teeth were immersed into melted wax (Horus; Herpo Produtos Dentarios, Petropolis, RJ, Brazil) to create a 0.2 mm- to 0.3 mm thick layer 2 mm apical to the cementoenamel junction. All specimens were embedded in autopolymerizing acrylic resin (Dencrilay, Dencril, SP, Brazil) in polyvinyl chloride cylinders. Following polymerization of the resin, the wax was washed away from the surface of the roots and the resin cylinder "sockets" with warm water for 2 s before injecting a polyether impression material (Impregum Soft; 3M ESPE, Paul, MN, USA) into the resin cylinders with an impression syringe. The teeth were placed back into their cylinder sockets. Each main group was then subdivided into three subgroups (A, B, and C, *n* = 8 each): Group A underwent neither a thermal nor mechanical cycling aging and served as the negative control group, whilst Groups B and C were subjected to 15,000 and 30,000 thermal cycles in water at temperatures ranging from 5℃ (± 2) to 55℃ (± 2) with a 1-min dwell time at each temperature and a transfer time of 5 s in a thermal cycling machine (MCT2-AMM2, Sao Paulo, SP, Brazil). For mechanical cycling, In order to simulate approximately 1½ and 3 years of clinical function [23] two different dynamic loading periods namely 3 × 10^5^ and 6 × 10^5^, were induced in a mastication simulator (Chewing Simulator, CS-4.8 professional line, SD Mechatronik GMBH, Westerham, Germany) with a nominal load of 49 Newton. The test parameters for the chewing simulator were modified to include: weight per sample of 5 kg; cycle frequency of 1.2 Hz;; horizontal movement of 0.3 mm; vertical movement of 6 mm; forward speed of 30 mm/s; rising speed of 55 mm/s; descending speed of 30 mm/s; and backward speed of 55 mm/s; and kinetic energy of 2,250 × 10–6 [24]. These tests ran at room temperature under 37 ± 3 °C water irrigation.

### Push-out test

2 mm from apical part of each root was sectioned and discarded, Then, each root was sectioned on an Isomet machine (Buehler, Lake Bluff, IL, USA) using a water-cooled diamond blade horizontally at 3 levels, apical, middle, and coronal, to obtain 3 sections, each measuring 2 mm in thickness. Digital caliper (Super caliper; Mitutoyo, Japan) was used to check and verify the thickness of each slice.

**Fig. 2:**
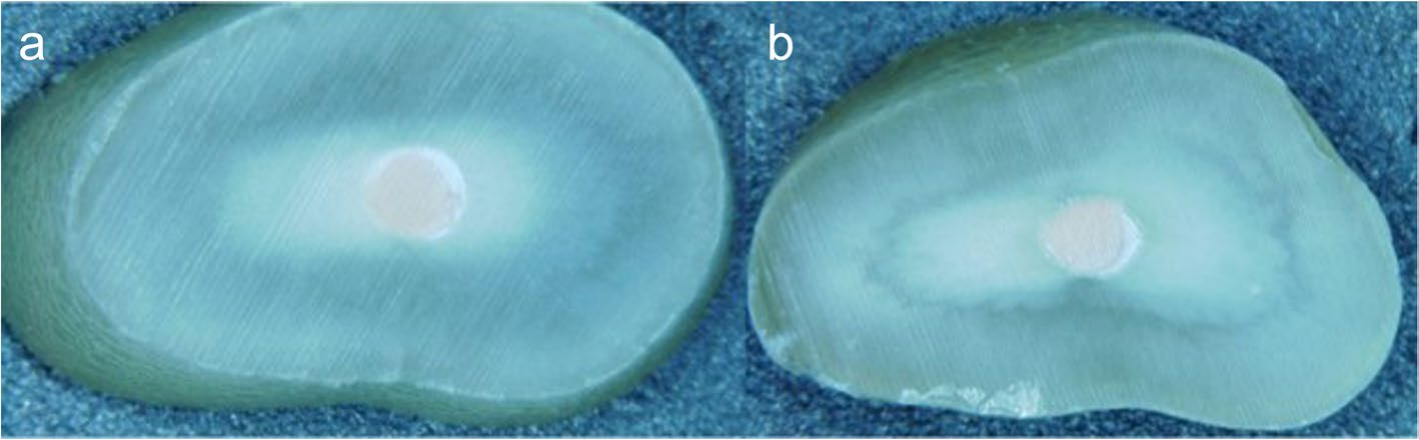
Inspection of the apical (**a**) and coronal (**b**) aspects of the slices following sectioning of the tooth horizontally

A light microscope (Zeiss Stemi SV6; Carl Zeiss, Jena, Germany) at × 8 magnification was used to investigate the coronal and apical aspects of the slices and ensure that the specimens were free from defects such as voids or cracks (Fig. 2). The specimens were set in a metal jig with a hole underneath to allow the root filling material to be expressed from the canal after dislodgement.

Each specimen's root filling was subjected to a vertical load applied in an apical to coronal direction. For this purpose, a universal testing machine (Instron, Canton, MA, USA) was equipped with a Ø 1.0 mm cylindrical plunger for coronal specimens, a Ø 0.8 mm plunger for middle specimens, and a Ø 0.6 mm plunger for apical specimens. During loading, the plunger only contacted the root filling. A loading speed of 1 mm/min was applied until dislodgement of the filling material. The highest force required to dislodge the core materials was measured in Newtons (N), and the POBS was determined from:

$$\mathrm{POBS}\left(\mathrm{MPa}\right)=\mathrm{Force }(\mathrm{N})/\mathrm{adhesion surface area }({mm}^{2})$$

The adhesion surface area was calculated from:

$$\mathrm{Adhesion surface area }({mm}^{2})=2\pi rh$$

where *r* is the root canal radius, $\pi$ is the constant 3.14, and *h* is the thickness of the root slice.

**Fig. 3:**
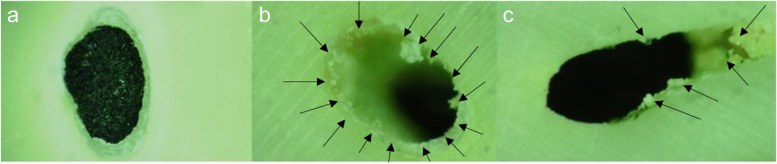
Digital microscopic examination of the samples at 100 magnification and various failure modes. Adhesive (**a**), cohesive (**b**) and mixed (**c**) failure types

The mode of failure was evaluated under a digital microscope (Hirox-KH7700, Hirox USA) at × 100 magnification (Fig. 3). The failures were categorized as adhesive failure (no material left on canal wall), cohesive failure (material present on entire canal wall), or mixed failure (material in patches on canal wall).

### Statistical analysis

All dislodgment resistance data were analyzed with statistical software (IBM SPSS Statistics v25.0, IBM Corp, NY, USA). The normality test of Shapiro–Wilk and Levene’s variance homogeneity tests were applied to the data and showed that the normality and homogeneity were satisfied (*p* < 0.05). Therefor repeated measures ANOVA was used to analyze the effect of the aging within root canal sealers; One-way ANOVA was used to compare sealers within different time periods. followed by aTukey post hoc comparisons test to reveal the existence of significant differences between the time and location among each sealer in push out bond strength test. An independent T-test was used to compare the mean of push-out bond strength for each group at different root canal thirds and times. Statistical analysis of the mode of failure of each sealer was performed using a Chi-square test.

## Results

Within location, Table 1 shows that at thermal–mechanical cyclic aging of 1½ and 3 years, AH Plus showed the highest bond strength in the apical third (1½ yrs = 4.70 ± 0.86; 3 yrs = 3.22 ± 0.48). While the lowest mean bond strength values were detected at the coronal root thirds (control = 1.10 ± 0.50; 1½ yrs = 0.91 ± 0.17; 3.0 yrs = 0.77 ± 0.22). Similarly, the highest bond strength for BioRoot RCS were in the apical third at control group (4.327 ± 1.109) and thermal–mechanical cyclic aging for 3 years (3.33 ± 0.87). In contrast, the lowest mean bond strength values were at the coronal root thirds (control = 1.53 ± 0.43; 1½ yrs = 0.26 ± 0.13; 3.0 yrs = 0.59 ± 0.19). Overall, as the number of thermal–mechanical cyclic aging increased to 1½ and 3 years the mean bond strength values were decreased accordingly for both sealers.

**Table 1:** Mean and SD within each sealer group, and between locations. MC (3 × 105) and MC (6 × 105) simulate approximately 1½ and 3 years of clinical function respectively

Sealer	Location	control	MC (3 × 10^5^)	MC (6 × 10^5^)	RM *P*-Value
AH Plus	Apical	2.301 ± 0.608^Aa^	4.704 ± 0.859^Ab^	3.219 ± 0.475^Ac^	0.000
	Middle	2.652 ± 0.521^Aa^	2.296 ± 0.72^Ba^	2.178 ± 0.233^Ba^	0.115
	Coronal	1.103 ± 0.495^Ba^	0.907 ± 0.165^Ca^	0.769 ± 0.215^Ca^	0.196
**ANOVA *P*-Value**		0.000	0.000	0.000	
BioRoot RCS	Apical	4.327 ± 1.109^Aa^	3.082 ± 0.694^Ab^	3.33 ± 0.866^Aab^	0.060
	Middle	3.222 ± 0.487^Aa^	1.456 ± 0.271^Bb^	1.159 ± 0.367 ^Bb^	0.000
	Coronal	1.533 ± 0.425^Ba^	0.258 ± 0.133^Cb^	0.587 ± 0.19 ^Cc^	0.000
**ANOVA *P*-Value**		0.000	0.000	0.000	

^a,A,b,B,C^Within same row, different superscript lowercase letters mean statistical difference between groups (*p* < 0.05). Within same column, different superscript uppercase letters mean statistical difference between groups (*p* < 0.05)

Between locations, no significant difference was found in bond strengths of the control at apical and middle thirds for AH Plus and BioRoot RCS (*p* > 0.05) (Table 1). Furthermore, after 1½ and 3 years of thermal–mechanical cyclic aging BioRoot RCS showed significantly lower mean push-out bond strength in all root thirds (4.32 ± 1.10, 3.22 ± 0.48, 1.53 ± 0.42) compared to control group obturated with BioRoot RCS.

Based on independent T-test Table 2, in control group the mean push-out bond strength values of AH Plus were lower than BioRoot RCS in apical and middle root thirds (2.30 ± 0.60 and 2.65 ± 0.52). However, after 1½ year of thermal–mechanical cyclic aging the mean push-out bond strength values of AH Plus were significantly higher than BioRoot RCS in all root thirds (control = 4.70. ± 0.85; 1½ yrs. = 2.29 ± 0.72; 3.0 yrs. = 0.90 ± 0.16). After thermal–mechanical cyclic aging for 3 years no significant difference was found among sealers at apical and coronal thirds (*p* > 0.05).

**Table 2:** An independent T-test to compare the mean of push out bond strength for each group at different root canal thirds and times

Location	Time	Sealer	Mean	Std. deviation	*P*-value
**Apical**	Control group	AH Plus	2.301	0.608	0.000
		BioRoot RCS	4.327	1.109	
	MC (3 × 10^5^)	AH Plus	4.704	0.859	0.001
		BioRoot RCS	3.082	0.694	
	MC (6 × 10^5^)	AH Plus	3.219	0.475	0.755
		BioRoot RCS	3.330	0.866	
**Middle**	Control group	AH Plus	2.652	0.521	0.040
		BioRoot RCS	3.222	0.487	
	MC (3 × 10^5^)	AH Plus	2.296	0.720	0.008
		BioRoot RCS	1.456	0.271	
	MC (6 × 10^5^)	AH Plus	2.178	0.233	0.000
		BioRoot RCS	1.159	0.367	
**Coronal**	Control group	AH Plus	1.103	0.495	0.083
		BioRoot RCS	1.533	0.425	
	MC (3 × 10^5^)	AH Plus	0.907	0.165	0.000
		BioRoot RCS	0.258	0.133	
	MC (6 × 10^5^)	AH Plus	0.769	0.215	0.094
		BioRoot RCS	0.587	0.190	

**Fig. 4:**
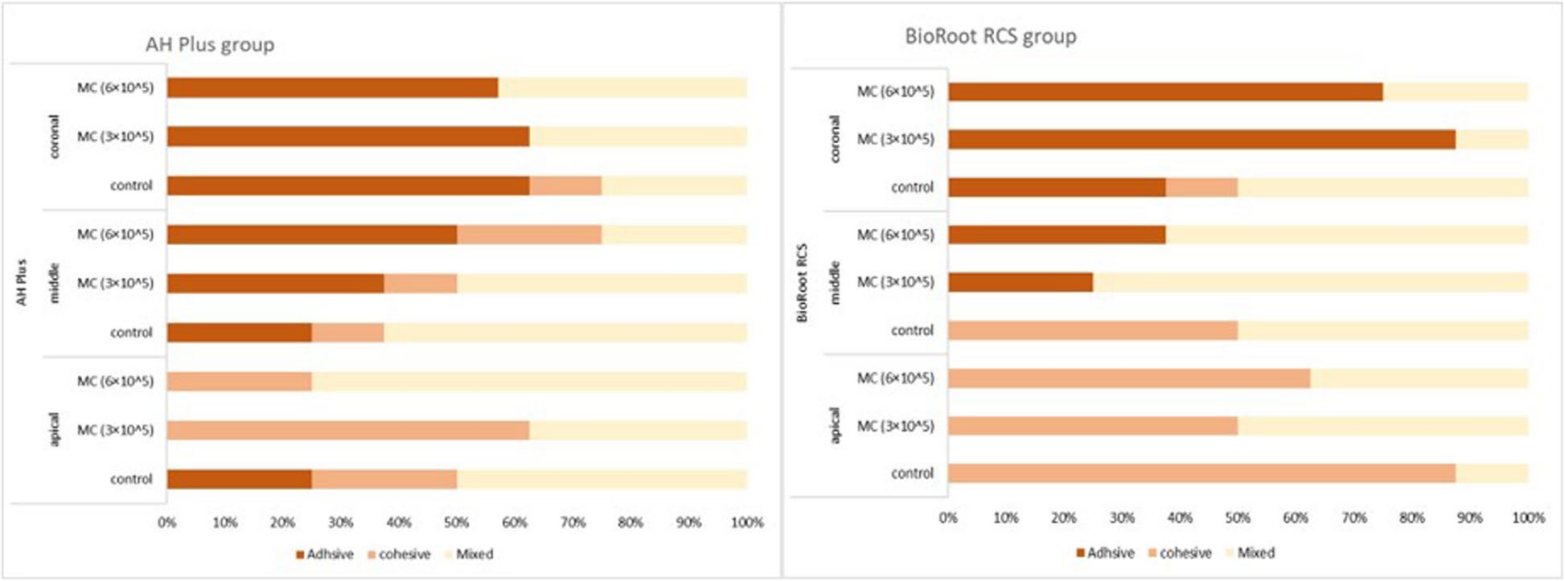
Distribution of failure modes in AH Plus and BioRoot RCS groups (in %)

The chi-square test revealed no association between groups and mode of failure (*p* > 0.05). Within each group, the Multiple Proportion test showed the distribution of failure was not equally likely. Mixed failure was the highest (Fig. 4).

## Discussion

According to Shipper, Ørstavik et al. [1], the material that adheres to the root canal walls will prevent the filling from dislodging. Improving the clinical performance and long-term service of root canal sealers is required to increase their retention to root canal walls. In vitro studies that replicate clinical situations should help in this regard. Thermal–mechanical cyclic aging was carried out to simulate the oral environment.

In the current in vitro study, 3 distinct pin sizes were selected in accordance with root canal thirds to be between 75 and 85% of the gutta-percha cone diameter, since the relationship between the pin diameter and the specimen diameter may affect the POBS [13]. To avoid premature debonding and sealer separation while slicing, 2 mm-thick slices were used [25].

To prevent lateral condensation and warm vertical compaction from potentially impacting the POBS, a single cone obturation using matching gutta-percha cones was used [26]. The push-out force was applied in the apical to coronal direction [27] as the root canal is tapered. If the loading had been in the coronal to apical direction, the validity of the results would have been affected.

In the current study, although both AH Plus and BioRoot RCS showed a decrease in the mean values of POBS after a simulated 3 years, AH Plus had significantly higher bond strength compared with BioRoot RCS after 1½ years in all root canal thirds. After 3 years, AH Plus exhibited nonsignificant differences in POBS (*p* > 0.05), comparable with BioRoot RCS in most root canal thirds. Thus, the null hypothesis of this study was rejected.

The authors are unaware of a previous study assessing the POBS of AH Plus or BioRoot RCS after thermal–mechanical cycling. Hence, because of the different methodologies used in the various studies, a direct comparison with other POBS studies was difficult. Nevertheless, the POBS of AH Plus sealer was primarily better than that of BioRoot RCS, which was consistent with POBS after aging reported previously [14–16, 28]. The improved bonding might be related to the covalent connections between the amino groups of the dentinal collagen and epoxy resin [29] so that the resulting polymer is heavily cross-linked and is thus rigid and strong [30] compared with the interaction of calcium silicates to dentin. Furthermore, the micromechanical tag-like attachment between the root canal wall and the calcium silicate-based sealer [31] may be affected by mechanical cycling, leading to the reduced push-out strengths observed in the BioRoot RCS group after artificial aging.

The lower POBS of BioRoot RCS compared with AH Plus after 1½ years could also be associated with the high solubility and porosity of this hydrophilic calcium silicate-based sealer [32] compared with the epoxy-based AH Plus and hygroscopic expansion reported for AH Plus; this expansion compensated for the resin-based sealer's polymerization shrinkage [33].

Unlike the current investigation, no statistically significant differences were reported in the bond strength values of AH Plus after 2 weeks or 3 months of incubation at 100% air humidity [34]. Another study reported that, compared with storage for just 1 week, the POBS of AH Plus sealer and other calcium-silicate sealers increased and were significantly higher after 4 weeks of incubation at 100% air humidity [27]. Moreover, Lin et al. (2021) compared the bond strength of BioRoot RCS with other methacrylate resin-based, calcium hydroxide-based sealers and MTA-based root canal sealer after thermocycle aging, BioRoot RCS and MTA-based root canal sealer showed increased bond strength with increased thermocycles[20]. However, either the thermal test alone or short period storage have been used in previous studies [27, 34, 35], whereas thermal–mechanical cyclic aging was used in the present investigation. Noteworthy is the finding that BioRoot RCS significantly outperformed AH Plus in terms of POBS in the control group. This is likely because BioRoot RCS released more calcium ions after setting, suggesting higher biomineralization in the dentin-cement interface and showing increased values of POBS, whereas AH Plus has a longer setting time because of the slow polymerization reaction of epoxy resin amines with a high molecular weight (bisphenol A and bisphenol F) where the conversion of monomers into polymers occurs gradually [20]. In addition, AH Plus appeared to contract during setting [36]. This finding was in accordance with several other studies that evaluated the impact of the presence or absence of a smear layer [17] or of using different dentin conditioning [37] and irrigation protocols [38] on the POBS of BioRoot RCS, which was better than that of AH Plus sealer.

AH Plus was reported to be nearly insoluble and epoxy resin sealants adhere to root canal dentin by creating a covalent link with dentinal collagen [39]. The degradation processes at the covalent interface between root dentin and epoxy resin root canal sealers may therefore explain the present results. However, the long-term creation of the calcium phosphate phase and the apatite layer differs depending on how calcium silicate-based sealers work [8]. This implies that the bioactivity and hydraulic nature of these sealers need not, as would be assumed, result in an increase in the sealer's POBS with time.

According to the results of this in vitro study, the apical third slices of AH Plus and BioRoot RCS exhibit higher POBS than the coronal and middle third slices after aging. The higher POBS was probably because of improved gutta-percha cone adaption, since increased pressure on the sealer improved dentinal tubule penetration [40]. Moreover, difficulty in removing the smear layer in the apical portion of the canal, which acts as coupling agent between dentin and BioRoot RCS might have a positive effect on the adhesion of BioRoot RCS to the root canal wall [41].

The coronal part of the root obturated with AH Plus and BioRoot RCS had the lowest POBS values (*p* < 0.05, probably because of increased sealer volume coronally than in the middle or apical regions. The increased sealer volume has more dimensional change while setting or dissolving during artificial aging [42]. AH Plus and BioRoot RCS predominantly showed mixed failure modes, consistent with previously reported findings [16, 43] Further comparisons with other investigations are not feasible since the experimental design in those studies either did not follow the approach used in the current study or did not describe the mode of failure. It can be assumed that while AH Plus and BioRoot RCS have a higher percentage of mixed failures, their connection to dentin is similar to that of gutta-percha. The lower percentage of cohesive failures provides proof of an attachment mechanism for calcium silicate-based and epoxy resin-based sealants to root canal dentin after thermal–mechanical cyclic aging.

Limitations of the present study include the use of distilled water as an immersion solution during mechanical cycling unlike the clinical situation. Whether different immersion solutions (phosphate-buffered saline, Hank’s balanced salt solution, or Dulbecco’s modified eagle medium) would impact POBS values is unclear. Thus, further investigation of the impact of other immersion solutions is needed to provide a more valid result.

Despite using rotary files with a consistent triangular cross-section geometry for preparing all specimens and employing an SU file size 25 taper (0.06) for achieving uniform shape and size of the prepared root canals, it would be valuable to investigate the influence of different cross-sectional root canal shapes on POBS results after undergoing thermal–mechanical aging.

## Conclusion

Within the limitations of this in vitro study, it can be concluded that thermal–mechanical cyclic aging affects the POBS of ETT in both AH Plus and BioRoot sealers. To obtain more consistent information that could be extrapolated to clinical practice, artificial aging should be used in laboratory studies.

## Data Availability

The data used to support the findings of this study are available from the corresponding author upon request.

## Abbreviations

ETT: Endodontically treated teeth
GP: Gutta-percha
POBS: Push-out bond strength
ANOVA: Analysis of variance
